# Screenathon 2.0: human–AI collaborative screening applied to patient-generated health data

**DOI:** 10.1038/s41598-026-45385-5

**Published:** 2026-03-22

**Authors:** Jonas Bergmann, Tiago Azzi, Rutger Neeleman, Kianush Monschau, Berke Yazan, Elena Jalsovec, Emily Westerbeek, Felix Weijdema, Jonathan de Bruin, Qixiang Fang, Rens van de Schoot

**Affiliations:** 1 Department of Methodology and Statistics, Faculty of Social and Behavioural Sciences, Utrecht University, Utrecht, The Netherlands; 2 Utrecht University Library, Utrecht University, Utrecht, The Netherlands; 3 Department of Research and Data Management Services, Information Technology Services, Utrecht University, Utrecht, The Netherlands

**Keywords:** Systematic literature screening, Human-AI collaboration, Patient generated health data, Large-scale systematic review, Computational biology and bioinformatics, Health care, Mathematics and computing

## Abstract

Systematic reviews are essential for evidence-based research, yet the traditional screening process is time-consuming and difficult to scale. Human-only screening can introduce inconsistency, while fully automated approaches employing Large Language Models often lack the contextual judgement required for complex decisions. To address this, we introduce a crowd-based screening methodology that integrates human expertise with adaptive machine learning. The methods have been applied in the context of a large EU project where experts from 27 collaborating partners jointly screened 5842 papers across eleven disease topics related to patient-generated health data in a span of 2 days. Post-processing played a central role in ensuring data quality, including topic reallocation, targeted full-text verification, and noisy-label filtering. This Screenathon resulted in 487 records being labeled as relevant and 6,463 records as irrelevant. The number of records screened per participant ranged from 3 to 2496, with a mean of 216.4 records per screener (*SE* = 95.19). Exploratory analyses using survey results indicated increased trust in AI-assisted systematic reviewing after the event, along with generally positive evaluations of usability. The current Screenathon demonstrates that crowdsourced human–AI collaboration requires thoughtful training and calibration, together with strong post-processing safeguards.

## Introduction

Systematic reviews are central to evidence-based research since they provide a transparent and comprehensive synthesis of existing studies on a given topic^2^. However, the rapid growth of scientific publishing has made the screening process increasingly difficult to manage^3^. Even when work is shared among teams, human-only screening remains time-consuming and vulnerable to inconsistency due to fatigue, subjective interpretation, and variation in domain expertise^4,5^. As research communities increasingly collaborate across disciplines, there is a need for screening workflows that are scalable, reliable, and feasible in practice.

We previously introduced the Screenathon Review, a time-saving and collaborative model designed to mobilize large groups of researchers to screen literature together^6^. The first Screenathon was implemented within the IMPROVE consortium, which comprises researchers, clinicians, and industry partners investigating patient-generated health data (PGHD)^7^. In this first Screenathon event, approximately 25 consortium members screened more than 12,000 title-abstract records over 4 days using shared inclusion criteria and real-time progress tracking. This approach demonstrated that coordinated collective screening can make screening large datasets in a short time frame possible and foster shared understanding within research teams. Yet, the process relied entirely on human judgment and therefore faced two core limitations. First, there were efficiency constraints, since manual screening scales poorly as evidence bases expand. Second, there was variability in labeling decisions, which can influence the quality and reproducibility of the review.

Recent work suggests that human-AI collaboration can address these limitations. Active learning systems can reprioritize records dynamically based on reviewer feedback, allowing likely relevant studies to appear earlier in the screening order while preserving human oversight^8–10^. Rather than replacing reviewers, these systems establish a human-in-the-loop workflow, where reviewers apply inclusion criteria, and the model continually updates its ranking of the remaining records. Evidence from comparative studies indicates that such hybrid approaches can yield decreases in the number of papers required to screen than either humans or models working independently^11–14^. In addition, a recent study further advances the field by systematically evaluating how automation tools can be implemented without compromising methodological standards. The authors aim to inform evidence-based recommendations for both systematic review authors and tool developers, with a particular focus on how automation can be integrated to optimize review efficiency while maintaining rigor. This work contributes to a growing body of guidance that moves beyond initial proof-of-concept evaluations toward the practical implementation of AI-assisted workflows^34^.

In traditional human-only screening, each record is independently reviewed by two human screeners, with discrepancies resolved through discussion or adjudication^6^. While robust, this approach becomes time-consuming and hard to manage as the number of records increases. AI-assisted dual screening retains the two-human model but incorporates a relevance-ranking algorithm to prioritize records based on real-time human feedback. This helps reviewers encounter likely inclusions earlier but maintains full human oversight of every decision^13,15^. More recently, crowd-based AI screening has been proposed, in which a shared AI model dynamically assigns records to a group of screeners (“a crowd of oracles”) based on their availability and collective labeling input^15^. This architecture uses active learning to continuously reprioritize records and distribute them intelligently across reviewers. Although promising in simulation, this model has not yet been used in real-world review settings. To address these gaps and extend our earlier Screenathon approach, we implemented a revised Screenathon that incorporated active learning as applied in the open-source screening software ASReview (2.0)^15^. In this approach, human reviewers serve as decision-makers whose labels provide real-time training signals, while the model reprioritizes records accordingly.

This study explored the feasibility of a collaborative screening approach combining human expertise with AI-assisted active learning. In the current paper, we report on the implementation and feasibility of this human-AI collaborative screening workflow in a large-scale PGHD literature review. We describe the procedure, introduce a training and calibration phase, and a post-processing framework to demonstrate how to maintain data quality when using AI-assisted workflows. Moreover, we compare screening outcomes and experiences to those of the earlier human-only Screenathon. We thereby propose that integrating active learning into collaborative screening can accelerate systematic review workflows while maintaining decision and participant engagement. In sum, we demonstrate how combining the Screenathon with active learning provides a scalable, reproducible, and user-friendly framework for large-scale, collaborative systematic reviews that fully leverage human–AI collaboration.

## Methods

### Overview

The methodology is structured across the following sections: First, the overall Procedure outlines the event’s schedule, training structure, and participant coordination. The data collection and preparation phase is detailed in the Literature Search section, which describes how the evidence base on patient-generated health data was updated, deduplicated, and allocated into 11 disease-specific projects. The mechanics of the AI integration and active learning loops are explained in the Human–AI Collaboration in the Current Screenathon and ASReview sections, while the practical deployment of this workflow by participants during the event is described in Screenathon v2 in Action. To ensure dataset completeness and accuracy after the accelerated AI-assisted screening, our rigorous post-event evaluation is detailed in Quality Check: Post-Processing. In parallel, the methods for evaluating user experience, trust, and motivation are outlined in the Questionnaire section, with all corresponding evaluation methods summarized under Statistical Analysis.

### Procedure

**Fig. 1:**
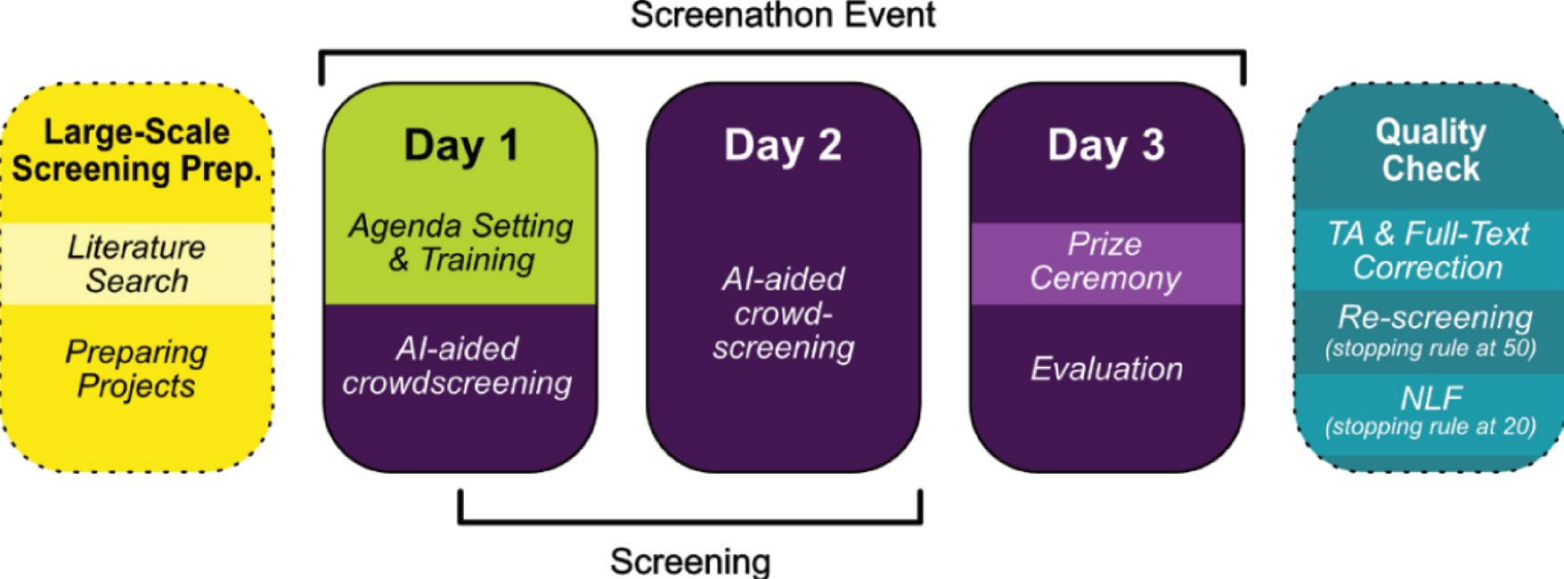
Overview of adapted screenathon review conducted at the IMPROVE plenary session. *Note*: The Screenathon consisted of four phases: (1) large-scale screening preparation, including literature search and creation of project-specific screening datasets; (2) group training and consensus building on screening criteria (Day 1); (3) collaborative AI-assisted title–abstract screening using active learning (Day 2); and (4) event closure and evaluation, followed by a structured post-screening quality check (Day 3 and post-event). The workflow was designed to combine shared domain expertise with real-time model updating to accelerate evidence screening across multiple disease topics.

We implemented the adapted Screenathon Review during the IMPROVE consortium plenary meeting held near Lecce, Italy (June 3–6, 2025). The procedure largely followed the structure outlined in Monschau et al.^5^, which involved screening preparation, group training, collaborative screening, and post-screening quality assessment (Fig. 1). Compared to the first Screenathon where all 12.473 records were screened (see ‘Workflow_Comparison’ on the OSF page^1^, , the current Screenathon screened the most likely relevant records as predicted by the machine learning algorithm. For further explanation, see section “ASReview”. We previously introduced a structured planning checklist for organizing Screenathon Reviews^6^. Here, we provide an updated version tailored to human–AI collaborative screening using active learning. A full checklist and printable planning template are provided on the OSF repository^1^.

Day 1 included both the training session and the first hour of screening. Because this Screenathon aimed to update rather than establish the evidence base, no calibration round was required; inclusion and exclusion criteria had already been defined and agreed upon during the earlier Screenathon. The training session consisted of a short presentation reviewing screening criteria with example title-abstract pairs, a handout summarizing decision rules, and open discussion for clarification, after which screening proceeded through iterative cycles of human labeling and AI-driven reprioritization. The participants were instructed to screen in their project until jointly a stopping rule of 50 consecutive non-relevant records was reached. On Day 1, participants screened for approximately one hour using ASReview 2.0.

Day 2 consisted of approximately 2.5 h of focused, AI-assisted collaborative screening. Participants could screen in a shared workspace or individually. Members of the organizing team were available throughout to answer questions and provide technical support. Participants were also encouraged to continue screening outside the scheduled session.

On Day 3, the Screenathon concluded with a brief closing session that included a small recognition activity. Participants and teams were acknowledged for different forms of engagement (e.g., consistency in screening, early participation, or balanced contribution within teams), and symbolic prizes were awarded to highlight collaborative effort and sustained motivation. Following the ceremony, attendees completed the post-event questionnaires, including an evaluation of the screening workflow and overall event experience.

### Literature search

Since a systematic review had already been conducted in the first Screenathon, detailed in Monschau et al.^6,18^, we updated the current search by applying a to-date filter for 2025 to capture newly published records. We conducted one database search targeting systematic reviews on PGHD and supplemented this with a broader search in OpenAlex to increase sensitivity. The search strategy was intentionally designed to maximize recall. The strategy comprised of a pre-registered literature search which was conducted in the PubMed, Embase, CINAHL and Scopus databases using search strings that incorporate MeSH terms and controlled vocabulary. The full, exact search strings for all databases are available on the Open Science Framework (OSF) repository (https://osf.io/hyuxw).

Eligibility criteria included: English publications between 2024 and 2025 with a persistent identifier such as a DOI; additionally, the title and abstract of the record had to be available^6,21^. For the title abstract screening, the inclusion criteria (see Table 1) were set as described in^21^.

**Table 1:** Literature screening inclusion criteria.

Criterion	Description
1. Study Type	Eligible are systematic reviews, literature reviews or meta-analytical publications. Importantly, relevant publications synthesize pre-existing primary research.
2. Content	Contains patient-generated health data (PGHD). PGHD are created by and captured from patients outside of clinical settings. Examples include but are not limited to self-report, sleep trackers, fitness trackers, continuous glucose monitors, and RFID-enabled implants. These data are commonly categorized as: Patient-reported experience measures (PREMs), patient-reported outcome measures (PROMs) and patient preference information (PPI).
3. Population	Included are studies examining adult human subjects (≥ 16 years old). Only those reviews and overviews where ≥ 80% of participants are adults should be included.
4. Condition Type	Studies examining both chronic and acute physical conditions are eligible.
5. Treatment	Study analyzes PGHD to any kind of treatment or medical recommendation. This includes not only medication taking, but also other health behaviors such as attending follow-up appointments, implementing lifestyle changes (e.g., avoiding certain foods, engaging in specific exercise) and using medical devices, among others.

*Note*. For a more detailed explanation of the procedure, as well as a case study of the title-abstract screening phase of this dataset, see Monschau et al^6^.

After deduplication and data cleaning, the combined searches yielded approximately 45,000 title–abstract records (2024–2025) of which 6950 papers were screened for relevance in the current Screenathon. These were then divided into eleven ASReview 2.0 projects, corresponding to the disease domains examined within IMPROVE (e.g., oncology, neurology, cardiovascular diseases). To promote early retrieval of relevant records during screening, each ASReview project was initialized with topic-specific inclusion priors. These priors consisted of full-text studies previously included in the first Screenathon’s systematic review for the same disease topic.

### Quality check

A brief topic allocation check was conducted during the Screenathon itself to ensure that reviewers were correctly assigned to their respective disease areas and that screening progress aligned with predefined topic structures. This real-time verification step helped identify minor allocation inconsistencies early on and supported smoother workflow coordination throughout the event.

The post-screening quality check began immediately after the event and continued for approximately six weeks, although this was mostly because multiple approaches to integrating and refine post-processing steps into the AI-assisted workflow were explored. This phase involved resolving conflicting labels, adjudicating unclear cases, and verifying the final set of included records, for a full overview, please see Table 2.

**Fig. 2:**
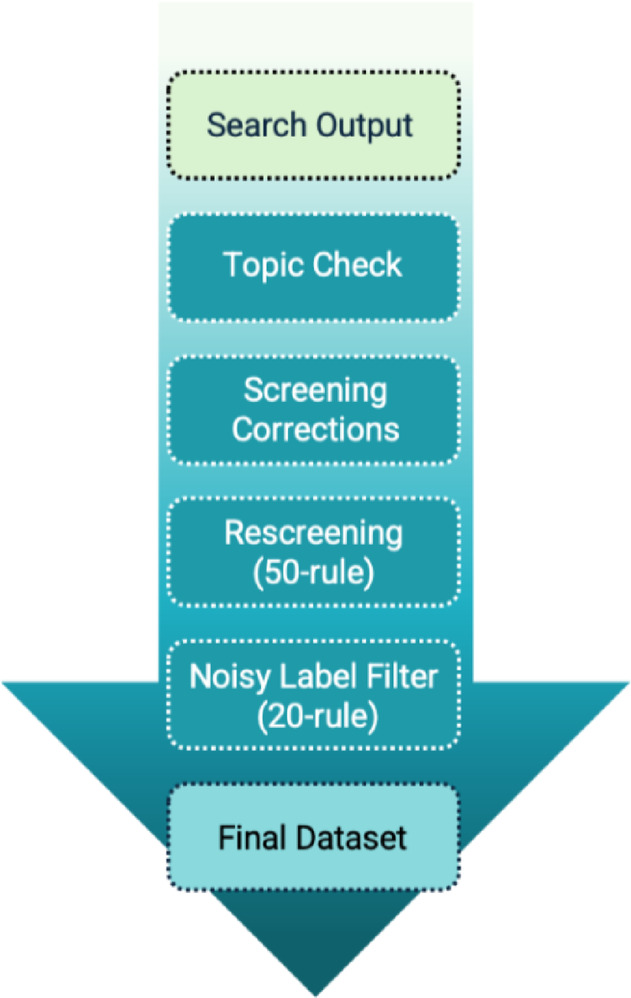
Post-processing workflow applied after the Screenathon. *Note*: The quality check involved four sequential stages. Topic allocation was first reviewed to ensure that records were assigned to the appropriate disease domains. Screening decisions were then verified and corrected where necessary. Each disease-specific pool was subsequently rescreened using a stopping rule of 50 consecutive irrelevant records to identify any remaining relevant studies. Finally, a noisy-label filter (20-record stopping rule) was applied to re-evaluate previously excluded records. The resulting dataset represents the final curated record set used for synthesis.

After the Screenathon, we conducted a structured post-processing workflow to ensure data quality, accuracy of topic assignment, and completeness of the final dataset. The workflow consisted of four sequential stages (Fig. 2). First, topic allocation was reviewed to verify that each record was assigned to the correct disease domain, and mislabeled records were reassigned where necessary. Second, title–abstract and full-text screening decisions were checked for consistency with the predefined inclusion criteria, and discrepancies were resolved through consensus review.

Third, each disease-specific record pool was rescreened using an a-priori stopping rule in which screening continued until 50 consecutive records were identified as irrelevant; any newly identified relevant studies were added to the dataset. Finally, we applied a noisy-label filtering procedure^16^ in which records previously excluded during screening were re-evaluated using a stopping rule of 20 consecutive irrelevant records^13^ All adjustments were logged to maintain transparency and reproducibility.

### Questionnaire

As in the first Screenathon^6^, participants were asked to report their confidence, motivation, and satisfaction throughout the event. The survey was deployed as an exploratory evaluation of participant experiences and attitudes toward AI-assisted screening. Considering the non-naïve sample and small group sizes, the statistical comparison are reported for descriptive purposes.

Immediately after the training session on Day 1, participants completed the first confidence and motivation questionnaire, indicating their self-perceived screening confidence and motivation on a slider scale (0 = not at all; 100 = extremely). At the beginning of Day 2, participants rated their satisfaction with their screening progress on Day 1 using the same scale and then completed a second confidence and motivation assessment. On Day 3, participants completed a more comprehensive feedback survey. In addition to general reflections on the Screenathon (e.g., what aspects were most helpful, and what could be improved), this survey focused on evaluating the new human–AI collaborative setup. Participants were asked about their experience using ASReview 2.0, what features or interface elements they would ideally like to see in an AI-assisted screening environment, and how much they trusted AI-aided systematic reviewing before and after participating in the Screenathon. Those who had taken part in both the first and current Screenathon were also asked to indicate which format they preferred. A complete overview of all questionnaire items is available in the OSF repository^1^.

### Human–AI collaboration in the current Screenathon

**Fig. 3:**
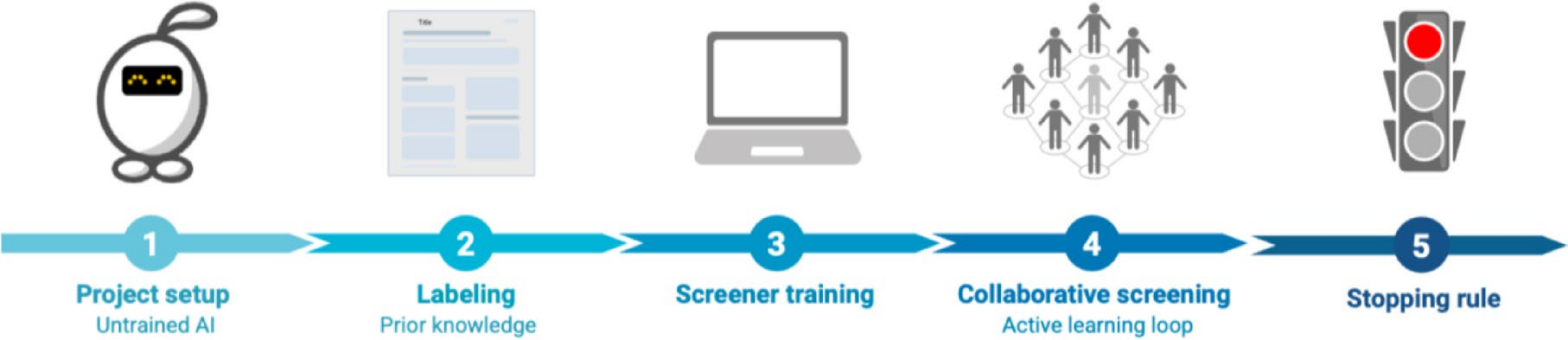
Human–AI collaborative screening workflow used in Screenathon v2. *Note*: The workflow consists of five sequential stages: (1) Project setup, in which an untrained model is initialized; (2) Warm-start labeling, where prior inclusion examples are provided to give the model initial context; (3) Screener training, during which participants receive instructions and screening criteria; (4) Collaborative screening in an active learning loop, where human decisions continuously retrain the model and reprioritize remaining records; and (5) Stopping rule, where screening ends after 20 consecutive non-relevant records are encountered.

**Fig. 4:**
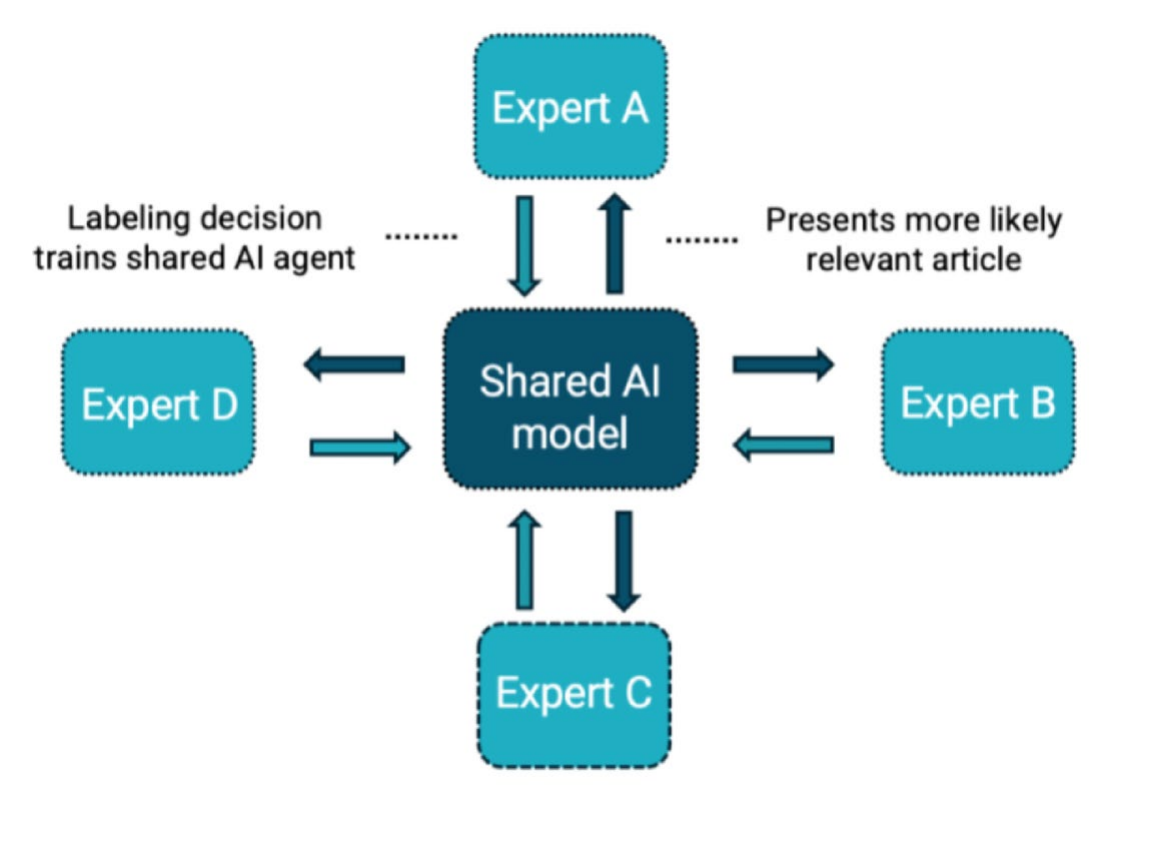
Multi-expert human–AI collaboration in ASReview v2. *Note*: Multiple screeners label records in parallel while contributing to a shared AI model. The model continuously reprioritizes unscreened records based on incoming labeling decisions and presents the next most likely relevant record to each screener. This setup supports coordinated large-group screening while maintaining a single, continuously updated model.

The current Screenathon differed from the first one in how AI support was integrated into the screening process (Supplementary Table 1). In this Screenathon, we used the active learning functionality of ASReview v2, meaning that the model continuously learned from each inclusion and exclusion decision made by participants^15^. After every labeling action, the model reprioritized the remaining records and placed those most likely to be relevant at the top of the screening queue. This created a human-in-the-loop collaboration^15,17^, where human judgments shaped the AI model, and the AI model guided what reviewers saw next (Fig. 3). In contrast, during the first Screenathon, all records identified in the search were screened. A small set of expert-labeled records was used to initialize the model, but record ranking was not updated as screening proceeded. As a result, the screening order did not adapt to the reviewers’ evolving decisions, and the process remained entirely human driven. In what follows, we will further detail the software used (Fig. 4).

### ASReview

#### Multi

ASReview LAB is an open-source, AI-assisted software platform designed to accelerate systematic literature reviews through active learning^9^. It follows open science principles with transparent, reproducible workflows while maintaining user data ownership and control. The platform uses machine learning to continuously learn from researchers’ screening decisions (inclusions and exclusions) and automatically prioritizes the remaining unscreened records, placing those most likely to be relevant at the top of the review queue.

ASReview v2 uses an iterative active learning cycle in which the model ranks unlabeled records and requests feedback from human screeners (“oracles”). Each cycle begins by transforming textual data into numerical representations (e.g., TF-IDF vectors or transformer-based embeddings), followed by training a classifier (such as a linear support vector machine). The trained model ranks the remaining unlabeled records by estimated relevance. Screeners then label the highest-ranked records, after which the model retrains on the expanded labeled dataset and reorders the remaining records accordingly. This cycle continues until a predefined stopping rule is reached^15^.

Version 2 introduces key advancements including support for collaborative screening with multiple expert reviewers (“a crowd of oracles”) working on a shared AI model, and a multi-agent system that can switch between different AI models (e.g., from fast general-purpose classifiers to domain-specific transformer models) as screening progresses (see fig. 4). The performance of ASReview has been tested in many simulation studies showing that the algoritms used by the tool always outperform random screening and can save up to 96% time depending on dataset and specific model, see the scoping review by Teijema et al.,^14^. ASReview v2 demonstrated a 24.1% reduction in prediction error compared to version 1 based on the SYNERGY benchmark dataset^15^. Simulation results using the labeled data from the first Screenathon show that, out of 12.473 studies, on average across 11 disease topics, only 450 papers (SD = 50) needed to be screened to identify all relevant records. These results provided the basis for the design of the current Screenathon.

### Warm-starting active learning with prior inclusions and exclusions

Active learning typically begins with an untrained model and a set of unlabeled records. Reviewers label records according to predefined inclusion criteria, and each decision serves as an immediate training signal. The model transforms text into numerical representations, learns patterns that distinguish relevant from irrelevant records, and updates its ranking of the remaining unscreened records accordingly.

Because the current Screenathon served to update an existing literature base rather than initiate a review from scratch, we warm-started the active learning model using prior knowledge from the first Screenathon. Specifically, we provided the model with an initial dataset of previously screened records and their labeling outcomes. This dataset consisted of 3,097 records identified across 11 database search iterations, supplemented with approximately 50,000 records retrieved from OpenAlex.

To initialize the active learning model, we considered several strategies for specifying inclusion and exclusion priors. Inclusion priors were straightforward: we used the full-text inclusions from the first Screenathon, as these records were confirmed relevant to their respective disease topics. For exclusion priors, however, several possible sources were evaluated (Table 2).

**Table 2:** Exclusion prior strategies.

Exclusion prior strategy	Description
Set A	All records from the previous review not included in any of the disease topics.
Set B	Records excluded during the title-abstract screening stage of the previous review.
Set C	Records assigned to the other ten disease topics but excluded during the full-text phase.
Set D	No exclusion priors.

We anticipated that Sets A and B might over-constrain the model by reinforcing older exclusion patterns, while Set C could offer higher-quality negative examples because those records had passed title–abstract screening but were excluded at full text. Set D, in contrast, would require the model to begin with inclusion-only priors, allowing initial negative labels to be provided directly by participants early in screening.

To evaluate these options, we conducted a small pilot test using one disease topic and three exclusion-prior configurations (Sets B, C, and D). For each configuration, we initialized the model and screened the first 15–20 records. Performance with Set D (no exclusion priors) was at least equivalent to the other configurations, while offering superior simplicity and flexibility. Based on this result, we selected Set D as the final initialization strategy.

During screening, each new inclusion or exclusion decision provided by screeners was used to update the u4 model in ASReview v2, which recalculated relevance scores and reprioritized the remaining records in real time. As the model adapted, screeners encountered fewer irrelevant records. Screening was discontinued once a stopping rule was reached, defined as a threshold number of consecutive non-relevant records.

### Screenathon in action

During the Screenathon, each disease topic was screened in its own ASReview project, with participants assigned to topic teams based on their expertise or interest. All screeners working on the same topic contributed to a single shared model for that topic, ensuring that each new inclusion or exclusion decision immediately informed the prioritization of remaining records for that specific disease domain.

Adopted from the full-text inclusions of the first Screenathon, the current Screenathon used inclusion prior to warming up each disease project. No exclusion priors were provided, consistent with the Set D decision described above. This approach allowed the model to begin with a minimal but reliable indication of relevance, while the negative examples were learned directly from participant labeling during screening. This setup was explained to participants during the group training session, so that screeners understood how their labeling decisions influenced the evolving model.

During collaborative screening, each labeling decision (relevant or not relevant) was fed back into the shared model, which then reprioritized the remaining unscreened records in real time. Because multiple screeners worked in parallel within each disease topic, the model benefited from the broader range of judgments and was able to converge more quickly on a stable relevance ranking.

Screening for each disease topic continued until the model reached the predefined stopping rule of 20 consecutive non-relevant records. At this point, the probability of identifying additional relevant studies in that topic was considered sufficiently low, and the screening session for that topic concluded.

### Statistical analysis

First, we will present the screening results: title-abstract and full text followed by quality check and post-processing. In the subsequent section, we report the survey results.

Survey data were analyzed using JASP^19^ and R^20^. For quantitative measures of confidence, motivation, satisfaction, and trust, we report means with 95% confidence intervals. Exploratory comparisons between Screenathon 1 and Screenathon 2 (e.g., Day 1 confidence and overall satisfaction) and pre- versus post-event trust ratings were examined using linear mixed-effect models (LMM), a one-way ANOVA, and paired samples *t*-tests, where appropriate. Post-hoc comparisons were corrected using Tukey’s Honestly Significant Difference (HSD) test. These analyses were intended to describe patterns in engagement and perceptions of AI-assisted screening rather than to support confirmatory hypothesis testing.

## Results

### Screening output

A total of 27 screeners participated in the second Screenathon. By the afternoon of the third screening day, 5,842 title–abstract records had been screened. The number of records screened per participant ranged from 3 to 2496, with a mean of 216.4 records per screener (*SE* = 95.19). From the extra screening day, 6,950 records had been screened. Of these, 487 records were labeled as relevant and 6,463 records as irrelevant.

**Fig. 5:**
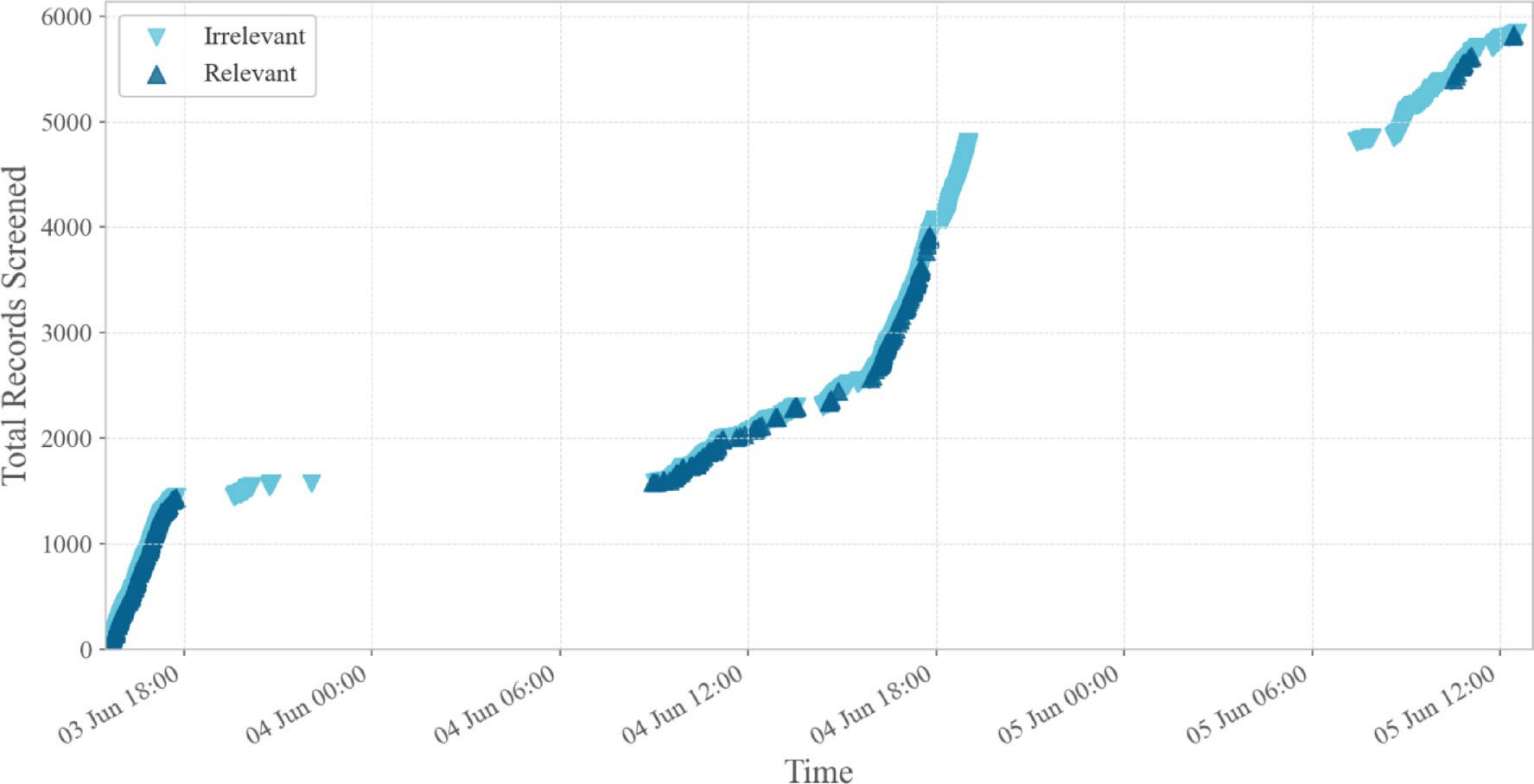
Cumulative screening progress during the Screenathon. *Note*: Cumulative number of records screened across the three screening days, separated by relevance label (relevant vs. irrelevant). Each point represents a labeling decision made by a screener in real time.

By the end of Day 3, six of the eleven disease-specific projects had reached the predefined stopping rule of 20 consecutive irrelevant records and were therefore considered complete (Age-related Macular Degeneration, Chronic Rhinosinusitis, Breast Cancer, Prostate Cancer, Severe Aortic Stenosis, and Atrial Fibrillation). The remaining five projects (Multiple Sclerosis, Cervical Cancer, Head and Neck Cancer, Coronary Artery Disease, and Heart Failure) continued into the post-processing phase for completion (see Fig. 5 for overview).

### Quality check: post-processing

During the correction phase, 88 misclassified records were removed across disease topics, and the full-text correction stage removed 206 records. Deploying the extra screening with a stopping rule of 50 (and max 1 h) included and 11 relevant full texts across the disease topics. 17 inclusions were identified after applying the NLF stopping rule of 20 consecutive irrelevant records. This resulted in a total of 221 inclusions. Table 3 shows the post-processing outcomes for each disease topic.

**Table 3:** Post-processing outcomes across disease topics.

Topic	Subtopic	Screenathon		Corrections		Extra screening		NLF	Total
		Total	Included	Topic	FT	Total	Included	Included	Included
Oncology	Prostate	460	45	– 14	– 14	53	0	0	17
Oncology	Breast	693	146	– 4	– 63	37	0	1	80
Oncology	Neck and head	97	25	– 4	– 7	104	5	0	19
Oncology	Cervical	130	8	– 5	– 1	90	3	0	5
Ophthalmology	Macular degeneration	89	8	0	– 7	14	0	0	1
Neurology	Multiple sclerosis	880	71	– 32	– 14	30	1	2	28
Chronic inflammation	Chronic Rhinosinusitis	3366	12	0	– 9	51	0	0	3
Cardio-vascular	Atrial fibrillation	228	27	– 25	– 2	0	0	0	0
Cardio-vascular	Coronary Artery	140	31	0	– 7	51	0	1	25
Cardio-vascular	Heart failure	319	45	0	– 18	54	2	13	42
Cardio-vascular	Severe aortic stenosis	548	69	– 4	– 64	27	0	0	1
Total		**6950**	**487**	**– 88**	**– 206**	**511**	**11**	**17**	**221**

*Note*: 6950 records screened; Included = based on title-abstract, the correction of those in post-process; followed by new screening. NLF = noisy label filter. FT = full-text.

### Daily screening progress

Figure 5 displays the cumulative screening progress across the event. The largest increase occurred on Day 2, with screening counts rising from 2515 to 5842 records. Notably, the steepest portion of the curve occurred during the afternoon session when many participants continued screening while gathered in informal shared spaces (e.g., around the pool), suggesting that flexible collaborative settings may support sustained screening engagement.

### Survey results

A total of 23 participants responded to at least one survey. After the training session on Day 1, the mean confidence in screening ability was 61.7 (*SD* = 26.6, *n* = 18).

Despite differences in confidence, the motivation to screen on Day 1 was high across participants (*M* = 82.6, *SD* = 18.5, *n* = 18). Satisfaction after completing screening on Day 1 was also high (*M* = 78.2, *SD* = 19.3, *n* = 15). Similar levels were observed for confidence and motivation prior to Day 2 (confidence: *M* = 77.1, *SD* = 15.2; motivation: *M* = 72.8, *SD* = 17.9; both *n* = 15) and for satisfaction after screening on Day 2 (*M* = 75.8, *SD* = 26.7, *n* = 12). Overall event satisfaction was high (*M* = 82.6, *SD* = 17.3, *n* = 13), and satisfaction with the gamification elements was particularly strong (*M* = 90.0, *SD* = 9.7, *n* = 12).

**Fig. 6:**
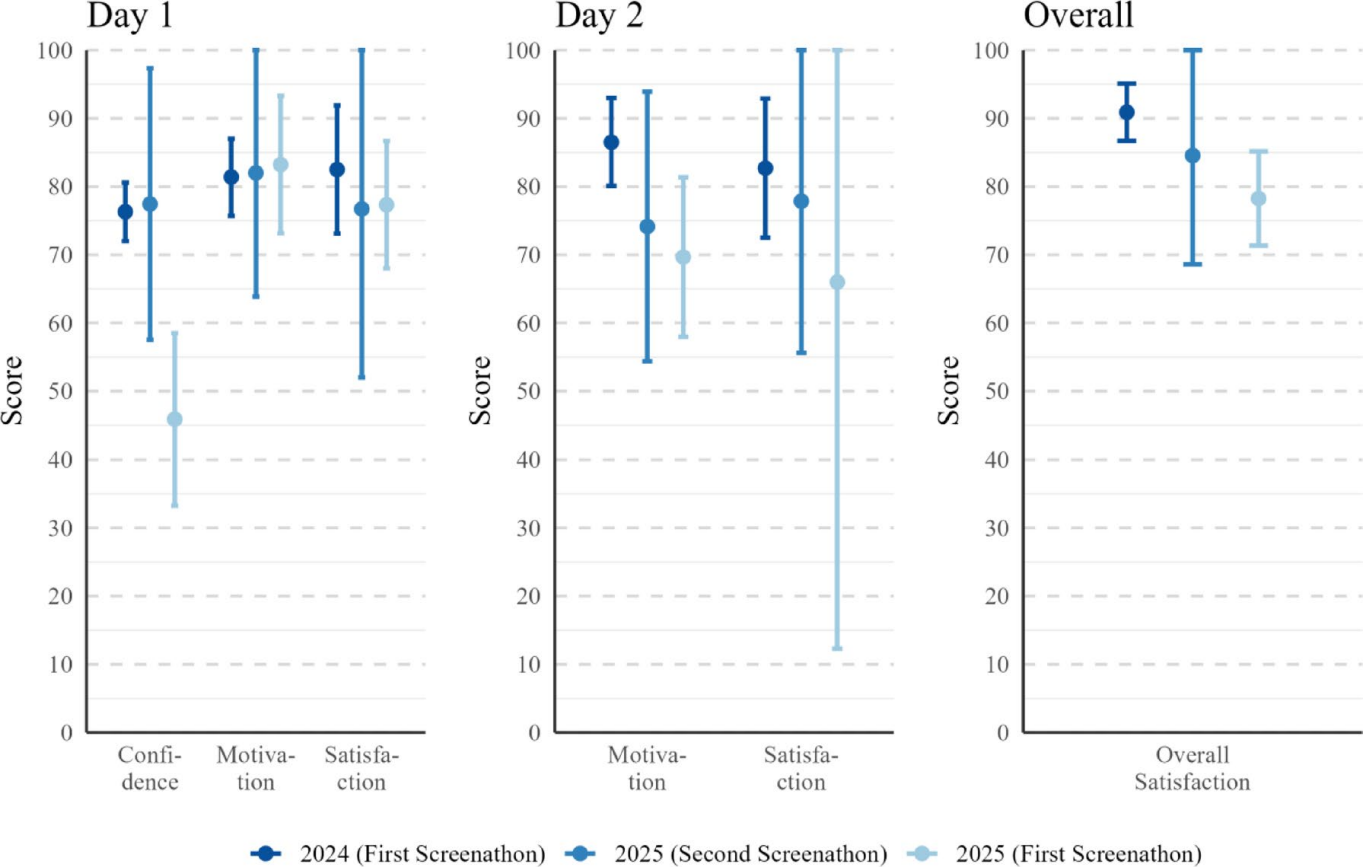
Comparison of quantitative survey outcomes across the 2024 and 2025 Screenathon events. *Note*: Mean scores (± 95% CI) are shown for confidence, motivation, and satisfaction immediately after Day 1 (left panel), prior to and after screening on Day 2 (middle panel), and overall satisfaction ratings (right panel).

Figure 6 compares overlapping survey items from the first Screenathon and the current Screenathon, with 2025’s Screenathon split by prior Screenathon experience. Visual inspection suggested differences in day 1’s confidence (between 2024 (first screenathon) and 2025 (first screenathon)), which was shown to be significant in the exploratory linear mixed-effects model (LMM): t(42) = 4.99, *p* < 0.001; and between 2025 (first screenathon) and 2025 (second screenathon), which was shown to be significant by the LMM: t(42) = – 4.23, *p* < 0.001. This suggests that prior exposure to the Screenathon format and inclusion criteria may have helped returning participants feel more prepared, whereas first-time screeners may have required additional or more scaffolded training. Day 2’s motivation (visually different between 2024 (first screenathon) and 2025 (first screenathon)) appeared insignificant in the exploratory LMM: t(30) = 2.38, *p* = 0.060, while overall satisfaction (between 2024 (first screenathon) and 2025 (first screenathon)) showed no significant difference in the exploratory one-way ANOVA (F(2,30) = 1.93, *p* = 0.163), indicating there was no main effect of prior screenathon experience on overall satisfaction scores.

**Fig. 7:**
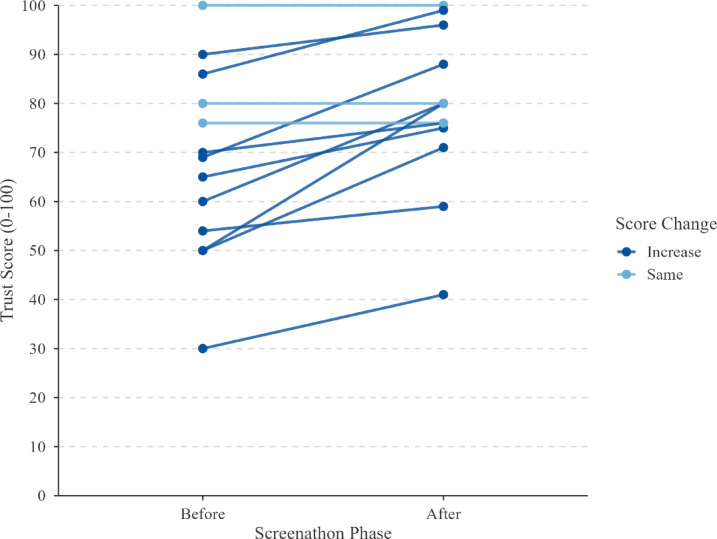
Individual trust scores in AI-aided systematic review methods, pre- and post-2025 Screenathon. *Note*: Each line represents one participant’s trust rating (0–100) before and after the event. Dark blue lines indicate higher trust scores on the scale shown to the right, whereas the light blue lines indicate the same scores before and after the Screenathon.

Finally, trust in AI-assisted systematic reviewing was assessed retrospectively before and after the Screenathon. Trust increased from a mean of 67.7 (*SD* = 19.2, *n* = 13) before, to 78.5 (*SD* = 16.2, *n* = 13) after the event. An exploratory paired t-test showed that this increase was significant (*t*(12) = 4.20, *p* = 0.001). This suggests that direct interaction with the human-AI screening workflow may have strengthened participants’ confidence in AI-assisted review methods (Fig. 7).

Table 4 summarizes the main themes identified from the open-ended survey responses (*n* = 14). Participants commonly highlighted the collaborative atmosphere, clear organization, and the flexibility of the screening setup as key strengths of the Screenathon. Suggestions for improvement primarily focused on refinements to screening procedures (e.g., clearer early guidance, more scaffolded examples) and practical logistics (e.g., timing, workspace setup). Overall, the qualitative feedback indicates strong engagement with the event and a generally positive experience, while also pointing to targeted areas for further optimization.

**Table 4:** Qualitative themes identified from participant survey responses.

Theme	*n*	Example responses
Overall atmosphere	5	“Environment,” “vibe,” “great experience”
Collaboration	2	“Team spirit,” “collaborative approach”
Organization	3	“Clear instructions,” “prizes”
Venue	2	“The venue,” “venue wasn’t bad either”
Flexibility	3	“Not bound to a location,” “freedom”
**Total positive responses**	**14**	–
Screening process improvements	6	“More time,” “exclude irrelevant papers,” “avoid plenary overlap”
Setup/logistics	2	“Better Wi-Fi,” “less warm weather”
No suggestions	4	“Nothing,” “I don’t know”
**Total improvement responses**	**11**	–

Screeners generally experienced ASReview v2.0 as intuitive and easy to use. Several participants highlighted its *user-friendliness* (*n* = 2) and *clarity of the interface* (*n* = 4), referring to the “simplicity,” “responsiveness,” and ease of understanding the UI. A few screeners also valued the *team-based setup* (*n* = 2), noting that being able to see who was screening together supported collaboration.

Suggestions for improvement primarily focused on enhancing efficiency and reviewer control. Screeners requested filtering options to prioritize specific records (*n* = 2) and additional functionality for managing screened papers and abstract text (*n* = 5), such as highlighting keywords, adjusting relevance labels more easily, and removing incomplete abstracts automatically. Some screeners (*n* = 3) did not identify any improvements needed. Regarding the dashboard, most screeners (58.3%) found its visualizations “somewhat intuitive”, while the remaining 41.7% described them as “extremely intuitive,” indicating generally positive usability with room for further refinement.

When asked to choose between review workflows (*n* = 13), AI-aided screening was preferred by most participants (*n* = 7; 53.8%). The Screenathon format itself was the next most preferred approach (*n* = 4; 30.8%), while only one participant expressed a (slight) preference for fully traditional screening. Comparisons among participants who attended both events (*n* = 8) were mixed: half preferred the 2024 screenathon, a quarter preferred the present event, and a quarter reported no preference, suggesting perceived differences in experience but no strong consensus shift.

Participants also indicated what information should be displayed during screening. All respondents favored title and abstract (100%), followed by inclusion/exclusion criteria (88.9%) and keywords (55.6%). Features such as DOI links, custom notes, or text-search tools were less frequently selected.

## Discussion

This study demonstrates that a human-AI collaborative screening workflow is operationally feasible in a large-scale consortium setting. The Screenathon represents a methodological shift from a purely human approach toward a structured human–AI collaborative workflow with a crowd of experts. The initial Screenathon setup (see also^6^ showed that coordinated human effort and a shared understanding of the screening tasks could efficiently process large evidence sets, in line with previous research on crowd screening^21^. However, it still assumed all identified records to be screened by experts and was constrained by fatigue and limited scalability. The current Screenathon setup addressed these issues by integrating active learning through ASReview v2.0, allowing the prioritization of records to update continuously based on a crowd of experts providing labels during a short period of time. This shifted the screening process from a static sequence of records to an adaptive system in which human decisions informed model behavior in real time.

Our study evaluated an integrated socio-technical system in which algorithmic components and human expertise are closely intertwined. The active learning model was iteratively refined through expert feedback, and its outputs were continuously supervised, interpreted, and validated by specialists. In addition, structured collaboration, prior shared training, topic expertise, and systematic quality assurance procedures played a central role in ensuring consistency and success of improved workflow. The resulting performance therefore reflects the interaction between computational prioritization and coordinated human judgment, rather than the contribution of the AI component alone. We accordingly conceptualize the findings as evidence of the potential and feasibility of a structured human-AI workflow rather than of an autonomous algorithmic intervention. The post-processing phase played a more central role in the current Screenathon than in the first. What had previously been a brief verification step became a structured quality assurance process involving topic reallocation, resolution of uncertain cases, and the application of a noisy-label filter^16^. This stage resulted in 221 additional inclusions, demonstrating that a strong post-processing workflow can safeguard completeness when live screening is accelerated by active learning^13^.

One of the key benefits of integrating AI features in systematic reviews is that it aims to address the issue of the increasing number of papers that need to be screened, leading to an immense workload and time investments when screening manually^34^. Active Learning has demonstrated its potential in reducing the number of screened records^35^, and recent investigations into the use of Large Language Models (LLMs) in systematic reviews show similar effects (e.g.^36^, . However, the efficiency of the AI-feature widely differs and strongly depends on the specific models selected^35^, the training data and the goals of the tool^34^. Moreover, studies have shown that AI-models, just like humans^5,37^, can produce errors in screening^37,34^. Besides a lack of access to AI tools or algorithms^34^, AI algorithms can remain a “black box” to the user, causing a lack of knowledge about why articles are presented or decisions are made^38^. To mitigate these risks, in the Screenathon Event, ASReview was used as an open-source software in which it is clear which models are used^39^.

The current Screenathon setup also offers insight into how researchers perceive and engage with AI in live screening environments. Participants reported significantly higher trust in AI-aided systematic reviews after the event compared to before. This suggests that direct interaction with active learning systems can support acceptance, particularly when users can observe how their labeling influences the model’s subsequent prioritization. These results are consistent with prior work indicating that hybrid human–AI approaches can achieve higher accuracy than either humans or models operating independently, in labeling tasks broadly^23^ and literature screening tasks specifically (e.g.^13^).

Participants generally described ASReview v2.0 as intuitive and easy to use. However, several noted a need for features that improve transparency and workflow navigation, including real-time filtering tools, clearer visualization of ranking progress, and stronger highlighting of key terms. These comments underscore that trust depends not only on performance but also on interpretability. Screeners must be able to understand how the model is making decisions if they are to rely on it during evidence-synthesis, as suggested by previous research^24^. The comparison between the first and second Screenathons reflects this shift. The first relied static model-based predictions, whereas the current approach introduced an adaptive prioritization process in which both model inference and human decision-making evolved together. This increased responsiveness likely contributed to the rise in trust and perceived usefulness, potentially heightening the sense of the AI model as a knowledge-producing entity^25^. At the same time, the feedback emphasizes that continued refinement of the user interface and transparency features is necessary to support widespread adoption and trust^26^. The results therefore point to both the potential and the present limitations of human–AI collaboration in systematic review workflows.

Besides ASReview, other tools have been developed over time to perform similar tasks. For instance, Rayyan^29^ is commonly employed in systematic literature screening (e.g.^27–29^). Similarly, DistillerSR^30^ is often used in, for example, medical sciences (e.g.^31,32^), as well as and abstrackr^27^. Unlike these tools, ASReview^9^ is both open-source, which could enhance trust in AI^33^, as screeners can see the models behind the predictions of relevant papers. Additionally, ASReview version 2^15^ features an integrated collaborative environment that supports crowdsourced screening, making it especially useful for the Screenathon Procedure. Unlike tools where reviewers must work in isolated project instances without real-time AI synchronization, ASReview allows multiple users to simultaneously train a shared model, making it especially useful for the Screenathon Procedure.

Quality check and post-processing served as the primary safeguard to ensure that the speed gained through human–AI collaboration did not come at the expense of accuracy. This is particularly important given that human screeners are also known to produce errors during screening^5,37^, underscoring the need for structured safeguards even in carefully designed workflows. Full-text verification was used to resolve ambiguous cases where title-abstract information alone was insufficient to determine eligibility^5^. Subsequently, each disease-specific record pool was rescreened using a predefined stopping rule, ensuring that screening did not end prematurely while avoiding unnecessary review of clearly irrelevant records^13,37^. Finally, the NLF^16^ was applied to re-examine records initially excluded during screening, allowing potential false exclusions to be recovered efficiently. This reciprocal process strengthened screening consistency, positioning post-processing as a central element of the human–AI collaborative workflow rather than a supportive correction step.

### Limitations

First, the survey evaluation was based on a relatively small sample, limiting statistical power and the generalizability of the findings. Participants were experienced domain experts from the IMPROVE consortium and therefore cannot be considered AI-naïve users. Their prior familiarity with systematic reviewing and exposure to AI-supported workflows likely shaped their baseline attitudes, expectations, and interaction patterns. As such, the results should not be interpreted as representative of broader reviewer populations, particularly novice screeners or interdisciplinary teams. Rather, the findings primarily demonstrate the feasibility of incorporating AI-assisted screening into established workflows among motivated experts in an in-person consortium setting. Additionally, because the inclusion criteria and disease topic structures had already been established during the first event, the training session in the current Screenathon was shortened. While this streamlined approach worked well for returning screeners, survey data indicated that new participants reported lower confidence following the training. This suggests that efficiency gains should be carefully balanced with adequate calibration time for first-time screeners. For example, future iterations could include extended onboarding sessions or separate training pathways tailored to different experience levels. Training should also be structured to formally assess whether prospective screeners reach consensus on training abstracts^22^. Future research should therefore include more diverse reviewer profiles and systematically examine whether experience level, disciplinary background, or disease group moderates engagement with AI-assisted screening. The exploratory statistical comparisons of confidence, motivation, and trust should be interpreted cautiously due to the risk of inflated Type I error. Differences were observed visually before testing, and the sample size was modest. Rather than serving as confirmatory evidence, these comparisons provide preliminary indications that returning screeners may feel more confident early in the process, and that participation may increase trust in AI-assisted screening.

Second, this study was exploratory and designed to demonstrate feasibility and workflow integration in a real-world collaborative screening setting rather than to provide a controlled performance evaluation. Although the results suggest that human, AI collaborative screening is operationally feasible, formal comparative evaluations are required to quantify performance metrics such as sensitivity, specificity, time savings, and error rates under controlled conditions. Furthermore, the exploratory statistical tests were not pre-registered, and pre-event trust in AI was self-reported retrospectively, meaning conclusions regarding increased trust must be framed cautiously.

Third, unlike the first event^6^ this Screenathon involved screening more records during the quality checking phase that already began during the event. This involved repeating the quality check process. As a result, much of the Screenathon occurred after the event rather than during it. This may be due to the shorter training session, which seemed to leave new screeners feeling less confident. Consequently, the quality of labels during the event likely declined because not all screeners had the same skill level. This led to an increased need for quality checks to correct more labels. For future Screenathons, greater focus should be placed on thorough training to improve the event and reduce the need for extensive, prolonged quality checks.

Fourth, the active learning configuration relied on warm-starting the model using prior screening data from the first Screenathon, as shown effective compared to a cold-start, see^40^. Specifically, the model was initialised with 3,097 previously screened records and their associated inclusion/exclusion decisions. While this reflects a realistic and large enough training set, warm-starting can bias retrieval toward earlier conceptualisations of the field and restrict exploration of novel or peripheral topics. Prior work has highlighted that warm-start approaches and exclusion prior strategies can affect model behaviour and retrieval outcomes, and that the choice of prior data should be carefully considered in active learning workflows to avoid anchoring effects and bias propagation see^41^. Fifth, regarding some records being automatically labeled as irrelevant because they were reviewed after reaching the stopping rule, we cannot exclude the possibility that some relevant papers remain in that list. At the same time, we needed pragmatic heuristics to make a live, two-day collaborative screening event operationally feasible. Because relying on heuristics instead of estimated stopping rules, carries the risk of leaving relevant papers unscreened or retaining incorrect labels^42^, we mitigated this risk by implementing an extensive post-processing quality check phase. To address this issue of stopping too early, we made efforts to avoid leaving any relevant record in the dataset by applying not only the NLF Procedure^16^, designed to correct mistakenly labeled relevant records, but also a relabeling process using a stopping heuristic of 50 consecutive records (discussed in refs^33,15^. We also verified that each included record was correctly assigned to its topic. Considering these factors, along with the limitations of human-only datasets^5^, our aim was to generate high-quality labels during the human-AI screening process context. This structured post-processing, which included topic reallocation, full-text verification, additional rescreening, and a Noisy-Label Filter (NLF) to catch false exclusions, served as our primary safeguard to ensure dataset completeness and accuracy. We acknowledge that sensitivity to the initial thresholds was not formally tested on this dataset, and we recommend that future work systematically examine how threshold choices affect recall.

## Conclusion

The current Screenathon demonstrates that systematic review workflows can be accelerated by integrating human expertise with adaptive AI-driven prioritization. By shifting from a static, human-only model to a dynamic human–AI collaborative system, the event achieved efficient large-scale screening while maintaining data quality.

## Data Availability

The data and materials are publicly available on the Open Science Framework (OSF) repository^1^: https://osf.io/hyuxw/files.

## Declarations

### Generative AI usage

Large language models were not used to generate text for the present paper. They were, however, utilized during editing to correct grammar mistakes and improve the writing style. Furthermore, they were used to help write R code to create figures. After using these tools, the authors reviewed and edited the content as needed and take full responsibility for the content of the publication.
